# Salivary Alpha Amylase Bronchial Measure for Early Aspiration Pneumonia Diagnosis in Patients Treated With Therapeutic Hypothermia After Out-of-hospital Cardiac Arrest

**DOI:** 10.3389/fmed.2022.880803

**Published:** 2022-05-13

**Authors:** Anis Moussali, Emi Cauchois, Julien Carvelli, Sami Hraeich, Fouad Bouzana, Audrey Lesaux, Mohamed Boucekine, Amandine Bichon, Marc Gainnier, Julien Fromonot, Jeremy Bourenne

**Affiliations:** ^1^Réanimation des Urgences, Timone University Hospital APHM, Marseille, France; ^2^Réanimation des Détresses Respiratoires et Infections Sévères, North University Hospital APHM, Marseille, France; ^3^Aix-Marseille University, School of Medicine—La Timone, EA 3279: CEReSS—Health Service Research and Quality of Life Center, Marseille, France; ^4^Department of Clinical Research and Innovation, Support Unit for Clinical Research and Economic Evaluation, Assistance Publique—Hôpitaux de Marseille, Marseille, France; ^5^Aix Marseille University, INSERM, INRAE, C2VN, Marseille, France; ^6^Laboratory of Biochemistry, Timone University Hospital APHM, Marseille, France

**Keywords:** cardiac arrest, alpha-amylase, aspiration pneumonia, acute lung injury, antibiotics

## Abstract

**Background:**

Aspiration pneumonia is the most common respiratory complication following out-of-hospital cardiac arrests (OHCA). Alpha-amylase (α-amylase) in pulmonary secretions is a biomarker of interest in detecting inhalation. The main goal of this study is to evaluate the performance of bronchoalveolar levels of α-amylase in early diagnosis of aspiration pneumonia, in patients admitted to intensive care unit (ICU) after OHCA.

**Methods:**

This is a prospective single-center trial, led during 5 years (July 2015 to September 2020). We included patients admitted to ICU after OHCA. A protected specimen bronchial brushing and a mini-bronchoalveolar lavage (mini-BAL) were collected during the first 6 h after admission. Dosage of bronchial α-amylase and standard bacterial analysis were performed. Investigators confirmed pneumonia diagnosis using clinical, radiological, and microbiological criteria. Every patient underwent targeted temperature management.

**Results:**

88 patients were included. The 34% (30 patients) developed aspiration pneumonia within 5 days following admission. The 55% (17) of pneumonias occurred during the first 48 h. The 57% of the patients received a prophylactic antibiotic treatment on their admission day. ICU mortality was 50%. Median value of bronchial α-amylase did not differ whether patients had aspiration pneumonia (15 [0–94]) or not (3 [0–61], *p* = 0,157). Values were significantly different concerning early-onset pneumonia (within 48 h) [19 (7–297) vs. 3 (0–82), *p* = 0,047]. If one or more microorganisms were detected in the initial mini-BAL, median value of α-amylase was significantly higher [25 (2–230)] than in sterile cultures (2 [0–43], *p* = 0,007). With an 8.5 IU/L cut-point, sensitivity and specificity of α-amylase value for predicting aspiration pneumonia during the first 2 days were respectively 74 and 62%. True positive and negative rates were respectively 44 and 86%. The area under the ROC curve was 0,654 (CI 95%; 0,524–0,785). Mechanical ventilation duration, length of ICU stay, and mortality were similar in both groups.

**Conclusion:**

In our study, dosage of bronchial α-amylase was not useful in predicting aspiration pneumonia within the first 5 days after ICU admission for OHCA. Performance in predicting early-onset pneumonia was moderate.

## Introduction

Out-of-hospital cardiac arrest (OHCA) is a frequent cause of ICU admission. Management of OHCA is clearly defined in specific guidelines (1). The high incidence rate of subsequent infectious complications is described in the literature (2, 3). Aspiration pneumonia is the most common complication, as a result of inhalation mechanisms during the early phase of CA and during cardio-pulmonary resuscitation (CPR) (4). Prophylactic antibiotic treatment is frequently initiated although benefit on mortality or neurological outcome has never been demonstrated (5–7). The recent work from François et al. (7) showed that prophylactic antibiotic treatment with amoxicillin-clavulanate in ICU patients after OHCA resulted in a lower incidence of infectious pneumonias. However, it had no effect on length of ICU stay, ventilator-free days, or mortality. Moreover, antibiotic overuse or misuse is a contributing factor to the spread of multidrug-resistant bacteria (8, 9). This issue should encourage judicious and proper use of antibiotics. The identification of a sensitive and specific biomarker for predicting aspiration pneumonia after OHCA seams necessary.

Alpha-amylase (α-amylase) is the major digestive enzyme in saliva (10). Its detection in bronchial secretions could be a marker of interest in aspiration pneumonia. Data suggests that elevated bronchial α-amylase value is associated with ventilator-associated pneumonia (VAP) (11). Samanta et al. (12) showed that mini bronchoalveolar lavage α-amylase concentrations increase in patients with VAP.

However, there is no data concerning the interest of bronchial α-amylase dosage in predicting the risk of developing aspiration pneumonia post-OHCA resuscitation.

The aim of this study is to determine the interest of bronchial α-amylase dosage in the early diagnosis of aspiration pneumonia, in ICU patients after resuscitation of OHCA.

## Materials and Methods

### Study Design

This is a prospective, observational study, conducted in an adult ICU in the University Hospital of Marseille (APHM, France), during a 5-year period (from July 2015 to September 2020).

### Patients

All adult patients admitted to ICU following OHCA (cardiac or respiratory etiology) were included. Targeted temperature management (TTM) was applied during the first 24 h, with continuous sedation and neuromuscular blockade. Exclusion criteria were patients under 18 years old, patients concerned by withdrawal of life-sustaining treatment decisions taken within a few hours after admission, and patients who died during the first 48 h after admission.

### Alpha-Amylase and Bronchial Sampling

A respiratory sampling was collected within the first 6 h after admission, using a protected specimen bronchial brushing (Combicath®, Prodimed) following a standardized procedure. This technique consists in inserting the device in the endotracheal tube until feeling it stop, then removing the spacer to allow the catheter to extend. A mini-bronchoalveolar lavage (mini-BAL) is then performed by injecting 40 mL of 0.9% saline solution, immediately aspirated using a sterile syringe. The sample collected was then transferred into 2 distinct sterile tubes, and immediately sent for biochemical and bacteriological analysis.

α-amylase dosage in the bronchial sampling was performed 24/7 in the biochemistry laboratory using a colorimetric enzymatic assay according to the International Federation of Clinical Chemistry (IFCC) recommendations (13). The α-amylase activity was detected without distinction of salivary or pancreatic isoforms. Bacteriological analysis and culture were performed on the same sample.

### Diagnosis and Treatment of Aspiration Pneumonia

Aspiration pneumonia was confirmed by the investigators retrospectively. According to the European Society of Intensive Care Medicine (ESCIM) (14) and the Infectious Disease Society of America/American Thoracic Society (IDSA/ATS) guidelines (15), required criteria were hyperleukocytosis > 10,000/μL or leukopenia <4,500/μL, presence of new or progressive radiological infiltrate or consolidation, associated with microbiologic confirmation in the respiratory sampling. Detection of mixed oral flora in the respiratory fluid was considered as a microbiologic confirmation of aspiration pneumonia in our study. Patients had to meet all 3 types of criteria (clinical, radiological, and microbiologic). Early-onset aspiration pneumonia was defined as pneumonia occurring during the 48 h following admission. Fever or hypothermia were not considered as diagnosis criteria, because of TTM influence on body temperature. Antibiotic treatment was left to physician discretion based on clinical arguments. No selective digestive decontamination or prophylactic antibiotic was realized. Only curative treatment was initiated by ampicillin-clavulanic acid, gentamicin was added if patient presented septic shock. The duration of treatment was 48 h without microbiological data or 5 days with microbiological data.

### Data Collection

Following data was collected during the first 3 days after admission and until day 5: OHCA characteristics (no flow and low flow time, cause), hemodynamic (heart rate, blood pressure, daily urine output, catecholamine use and dose) and respiratory variables (ventilator parameters, ratio of arterial oxygen pressure and fraction of inspired oxygen (P/F) several times a day), and body temperature. Microbiologic results, severity scores SOFA an SAPS2, as well as biochemistry results were collected (white blood cell count, procalcitonin (PCT), arterial blood gas analysis, arterial lactate, serum troponin at admission, H+6, and H+12). We also documented all the procedures underwent by patients: coronary angiography, pericardial drainage, extracorporeal life support, anti-infective treatments. The occurrence of post-cardiac arrest syndrome with myocardial and/or microcirculatory dysfunction was specified. Complications such as multiple organ failure, additional cardiac arrest, hemorrhagic shock, required renal replacement therapy (RRT) or prone position were collected. For each patient, the length of mechanical ventilation and ICU stay, as well as mortality rate were collected.

### Statistical Analysis

Qualitative variables, resumed by counts and percentages, are compared using Khi^2^ or Fisher exact test. Quantitative values are presented with medians and interquartile ranges (IQR). They are compared using Mann and Whitney test. Sensitivity, specificity, and predictive values of α-amylase were determined. La ROC (Receiver Operating Characteristic) curve was established to determine the best cut-point value for predicting aspiration pneumonia. We used SPSS software version 20 for statistical analysis. The threshold for statistical significance was defined for *p* < 0.05. The number of needed was calculated on the only value reported a threshold of 125 IU/ml to predict the risk of aspiration pneumonia. If we assume a difference of 20 ± 30 IU/ml, 37 subjects per group would be necessary with a power of 80% and an alpha risk of 5%, i.e., 64 subjects. Hundred patients will be included to overcome any inclusion problem.

### Ethical Considerations

This study was accepted by the Committee for the Protection of Persons of Marseille (N°2016A0119744AEC). The Commission on Data Processing and Freedom was notified (N° CNIL1994062v0). The study was registered in the Clinical Trial database (NCT 03007862). All patients or relatives received an information note, and a written consent was obtained.

## Results

### Population

From July 2015 to September 2020, 88 patients were included. Clinical and biological data, as well as treatments received are presented in Table 1. Cardiac disease (arterial coronary disease, arrhythmia, pulmonary embolism) was the predominant cause of OHCA (61%). Hypoxia was found in 26% of the situations. Median no flow time was 2 mins (IQR 0–10), and median low flow time was 15 mins (IQR 10–20). A coronary angiography was performed immediately after admission in 47% of cases. 57% of patients received prophylactic antibiotic treatment on their admission day. Among them, 50% received amoxicillin/clavulanic acid and 34% received a combination of amoxicillin/clavulanic acid—gentamicin. Median maximal temperature on day 1 was 36°C (IQR 35–37). Post-cardiac arrest syndrome with hemodynamic dysfunction occurred in 25% (*n* = 22 patients) of cases on day 1, 21% (*n* = 9 patients) on day 3. ICU mortality rate was 50% (Table 2).

**Table 1:** Clinical characteristics of patients at Intensive Care Unit (ICU) admission.

	**Global population (*n* = 88)**	**No aspiration pneumonia (*n* = 58)**	**Aspiration pneumonia (*n* = 30)**	***p***
Age (years)	59 (46–68)	59 (45–68)	59 (47–68)	0.979
Gender: male (*n*, %)	60 (68)	39 (67)	21 (70)	0.792
Weight (Kg)	80 (65–92)	80 (70–92)	76 (65–100)	0.772
No flow duration (minutes)	2 (0–10)	2 (0–10)	5 (0–9)	0.707
Low flow duration (minutes)	15 (10–20)	15 (10–20)	15 (12–21)	0.224
*Cardiac arrest etiology*				0.731
Cardiac etiology (*n*, %)	54 (61)	34 (59)	20 (61)	
Hypoxia (*n*, %)	23 (26)	17 (29)	6 (20)	
Subarachnoid hemorrhage (*n*, %)	2 (2)	1 (2)	1 (3)	
Hypokaliemia (*n*, %)	1(1)	1 (2)	0 (0)	
Hemorragic shock (*n*, %)	2 (2)	2 (3)	0 (0)	
Electrocution (*n*, %)	1(1)	0 (0)	1 (3)	
No etiology (*n*, %)	5 (6)	3 (5)	2 (7)	
*Emergency treatment*				0.936
Coronarography (*n*, %)	41 (49)	29 (58)	16 (53)	
ECMO (*n*, %)	2 (2)	2 (3)	0 (0)	
Temporary pacemaker (*n*, %)	1 (1)	1 (2)	0 (0)	
Pericardial drainage (*n*, %)	1 (1)	1 (2)	0 (0)	
Antibiotic therapy at admission (*n*, %)	50 (57)	33 (57)	17 (57)	0.984
SOFA score at H24	10 (8–11)	10 (7–11)	10 (8–12)	0.340
SAPS II score	64 (56–75)	64 (55–71)	69 (58–80)	0.079
SOFA score at H48	8 (6–11)	8 (6–11)	10 (7–12)	0.199
Bronchial amylase level at admission (IU/L)	3 (0–90)	3 (0–61)	15 (0–94)	0.157

*No flow represents cardiac arrest delay without cardio pulmonary resuscitation (CPR). Low flow represents CPR duration*.

*ECMO, Extracorporeal membrane oxygenation; SOFA, Sequential organ failure assessment; SAPS II, Simplfied Acute Physiology Score II*.

*Values are presented as medians +/– Inter Quartile Range (IQR)*.

**Table 2:** Evolution of patients' clinical characteristics from day 1 to 3.

	**Global population (*n* = 88)**	**No aspiration pneumonia (*n* = 58)**	**Aspiration pneumonia (*n* = 30)**	***p***
*Clinical characteristics on day 1*				
Body temperature (°C)	36 (35–37)	36 (35–37)	36 (35–37)	0.412
White blood cell count (G/L)	16 (13–21)	16 (12–20)	17 (14–22)	0.500
PaO_2_/FiO_2_ ratio	213 (130–267)	203 (128–265)	238 (146–321)	0.233
Arterial lactate level (mmol/L)	4 (2–8)	4 (2–9)	4 (2–7)	0.682
PCAS (*n*, %)	22 (25)	9 (16)	13 (43)	0.005
*Clinical characteristics on day 2*				
Mechanical ventilation (*n*, %)	84 (97)	56 (98)	28 (94)	0.272
Body temperature (°C )	38 (37–38)	38 (37–38)	38 (37–38)	0.683
White blood cell count (G/L)	14 (10–19)	13 (10–19)	15 (13–20)	0.192
PaO2/FiO2 ratio	214 (155–283)	213 (154–278)	215 (153–290)	0.834
Arterial lactate level (mmol/L)	2 (1–3)	2 (1–2)	2 (1–3)	0.313
PCAS (*n*, %)	18 (21)	7 (12)	11 (37)	0.008
*Clinical characteristics on day 3*				
Mechanical ventilation (*n*, %)	67 (81)	42 (76)	25 (89)	0.158
Body temperature (°C )	38 (37–38)	38 (37–38)	38 (38–38)	0.832
White blood cell count (G/L)	13 (10–17)	13 (10–17)	13 (9–17)	0.952
PaO2/FiO2 ratio	254 (172–298)	260 (189–303)	243 (165–283)	0.414
Arterial actate level (mmol/L)	1 (1–2)	1 (1–2)	1 (1–2)	0.815
PCAS (*n*, %)	9 (11)	2 (4)	7 (26)	0.005
PCT (μg/L)	0,7 (0,2–2,9)	1,0 (0,2–3,4)	0,6 (0,2–1,5)	0.063
Mechanical ventilation duration (days)	5 (3–8)	5 (3–8)	6 (4–9)	0.380
ICU hospitalization duration (days)	6 (4–9)	6 (4–9)	6 (4–9)	0.975
ICU mortality (*n*, %)	44 (50)	27 (47)	17 (57)	0.368

*Values are presented as medians +/– Inter Quartile Range (IQR)*.

*PCAS, Post Cardiac Arrest Syndrome; PCT, serum procalcitonine*.

### Aspiration Pneumonia

After clinical, radiological, and bacteriological data analysis, aspiration pneumonia diagnosis within the first 5 days was established for 30 patients (34%). The 17 (56%) pneumonias occurred during the first 48 h.

**Figure 1:**
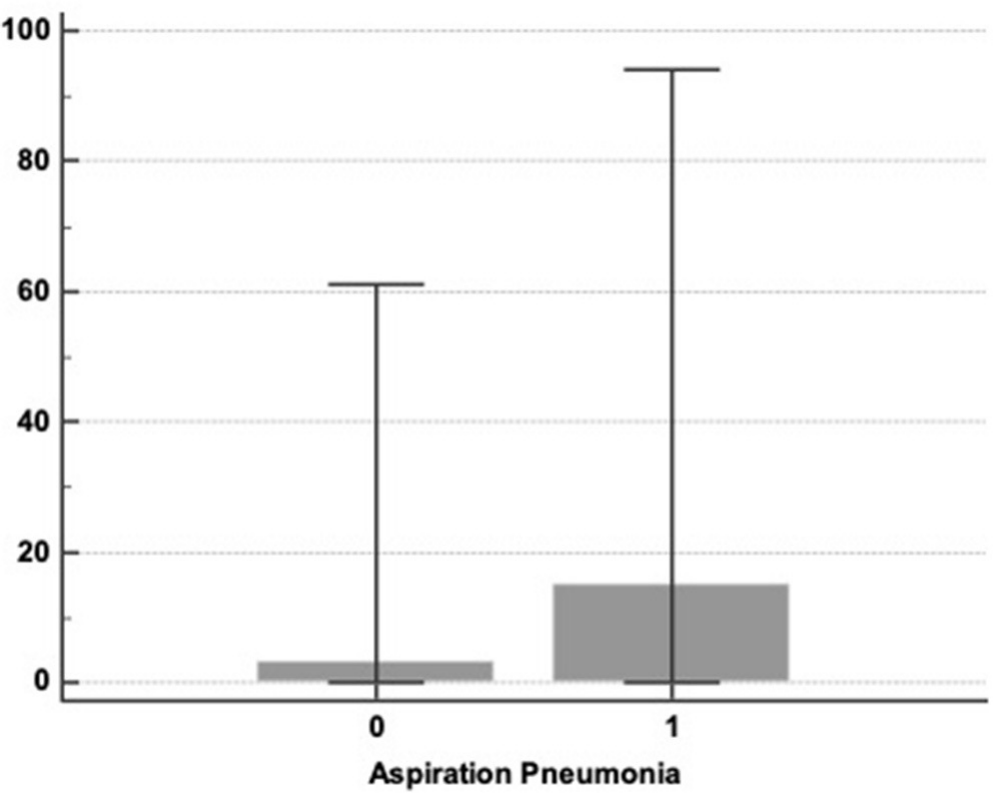
α-amylase levels in mini-BAL at admission and aspiration pneumonia represented by box plot 25–75: 0: no aspiration pneumonia; 1: apiration pneumonia [3 (0–61) vs. 15 (0–94) *p* = 0.157].

We found no significant difference concerning α-amylase value in the mini-BAL between the two groups, with and without pneumonia [3 (0–60) vs. 15 (0–130)—*p* = 0,157—Figure 1].

**Figure 2:**
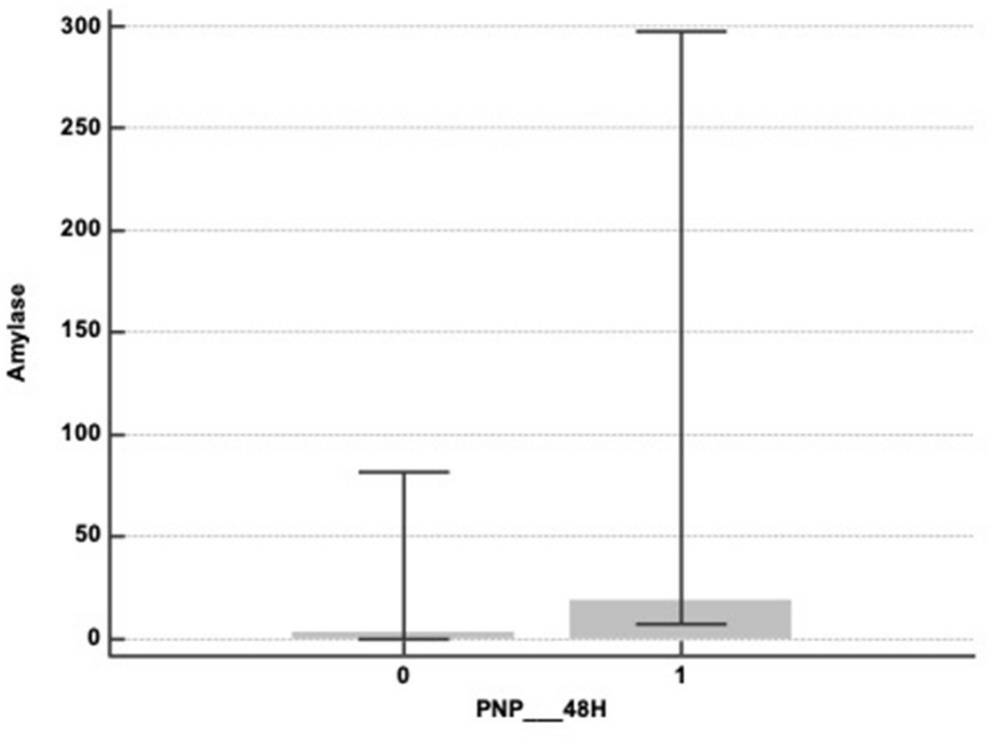
α-amylase levels in mini-BAL at admission and early aspiration pneumonia (within the first 48 h) represented by box plot: 0: no early aspiration pneumonia; 1: early apiration pneumonia [19 (7–297) vs. 3 (0–82)—*p* = 0.047].

However, we found a significant difference concerning bronchial α-amylase values between patients who developed early aspiration pneumonia (≤48 h—*n* = 17) and the others [19 (7–297) vs. 3 (0–82)—*p* = 0,047—Figure 2].

**Figure 3:**
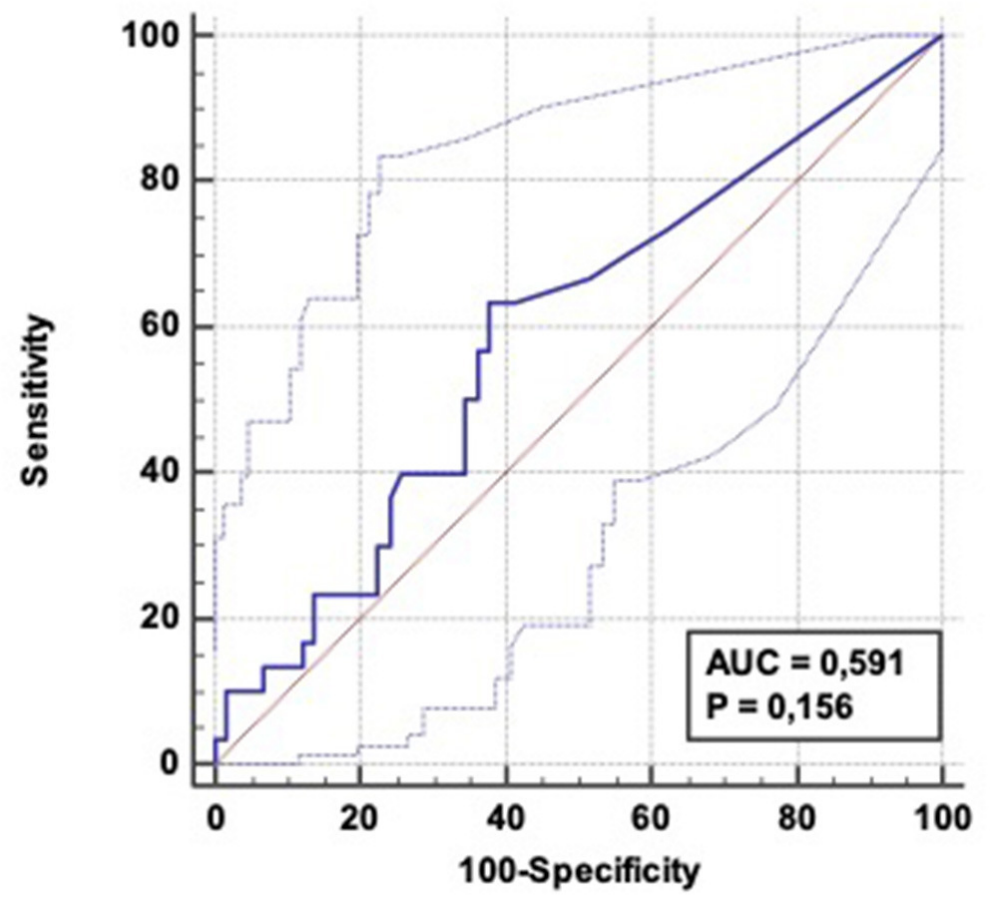
ROC curve: Diagnostic ability of α-amylase level to predict aspiration pneumonia occurrence within 5 days following OHCA admission. AUC = 0.591 (CI 95%: 0.464–0.717); Positive LR = 1.67 (CI 95%: 1.1–2.6); Negative LR = 0.59 (CI 95 %: 0.4–1.0).

The area under the curve (AUC) was 0.591 (CI 95%: 0.464–0.717) (Figure 3). For an 8.1 IU/L cut-point, the sensitivity and the specificity of α-amylase for predicting aspiration pneumonia within the first 5 days were respectively 63% (CI 95%: 44–80) and 62% (CI 95%: 48–74). True and false positive rates (TPR and FPR) were respectively 46% (CI 95%: 31–62) and 77% (CI 95%: 62–87).

**Figure 4:**
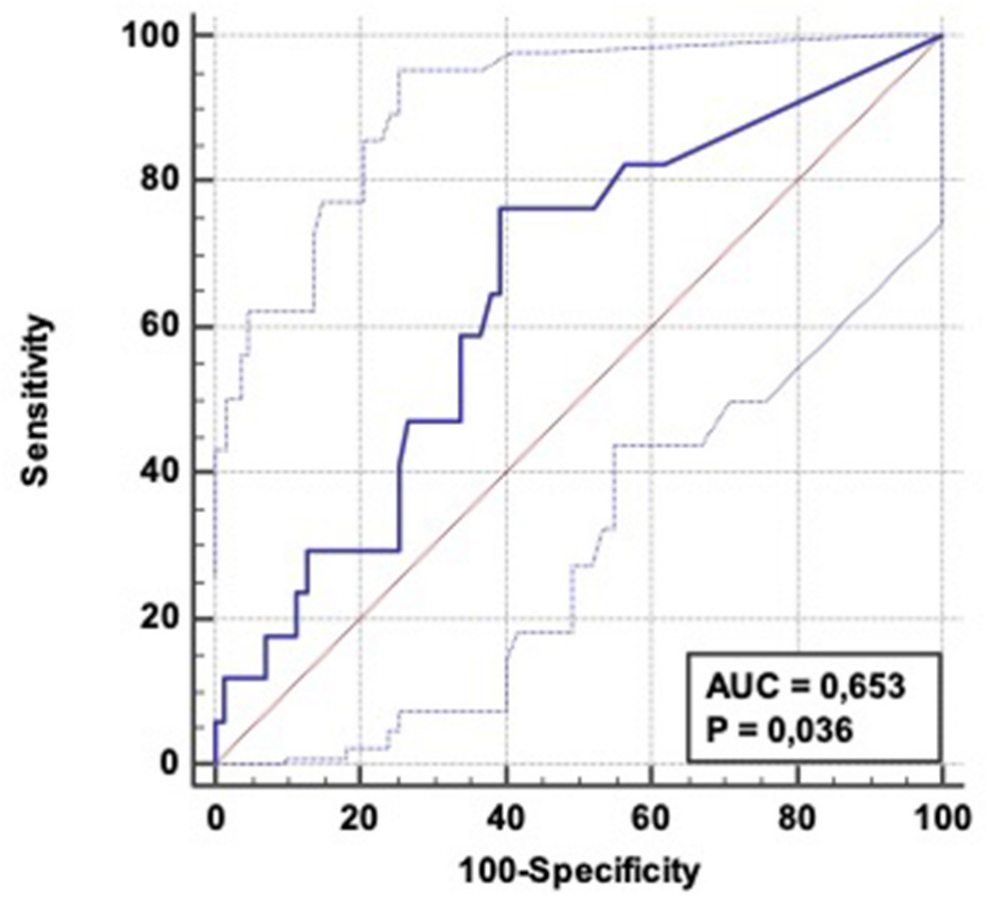
ROC curve: Diagnostic ability of α-amylase level to predict aspiration pneumonia occurrence within 48 h following OHCA admission. AUC = 0.653 (CI 95%: 0.524–0.0.785); Positive LR = 1.94 (CI 95%: 1.3–2.9); Negative LR = 0.39 (CI 95 %: 0.2–0.9).

Using the same α-amylase cut-point, values of sensitivity/specificity/TPR/FPR for predicting early aspiration pneumonia (48 h—*n* = 17) were respectively 76.5% (CI 95%: 50.1-−93.2), 61% (CI 95%: 48.3–72), 32% (CI 95%: 24–41), and 91.5 % (IC 95%: 81.7–96), with an AUC of 0,653 (CI 95%: 0,524-−0,785) (Figure 4).

Aspiration pneumonia occurrence did not significantly impact median mechanical ventilation duration (5 days, IQR: 3–8), ICU stay (6 days, IQR: 4–9), or ICU mortality (44 patients-−50%).

### Microbiologic Data

Table 3 shows the different microorganisms identified. The most common was *Staphylococcus aureus* (29%), followed by *Haemophilus influenza* (13%), and *Streptococcus pneumonia* (10%). Mixed oral flora was found in 26% of our samplings.

**Table 3:** Microbial identification in ICU admission bronchial sampling.

**Bacterial identification**	n (%)^*^
Gram positive	
*Staphylococcus aureus*	9 (29)
*Streptococcus pneumoniae*	4 (9.7)
*Other streptococcus*	2 (6.5)
Gram negative bacillus	
*Haemophilus influenzae*	4 (12.9)
*Escherischia coli*	2 (6.5)
*Klebsiella pneumoniae*	1 (3.2)
*Klebsiella oxytoca*	1 (3.2)
*Serratia odorifera*	1 (3.2)
*Serratia liquefaciens*	1 (3.2)
*Proteus mirabilis*	1 (3.2)
*Enterobacter cloacae*	1 (3.2)
*Haemophilus parahemolyticus*	1 (3.2)
*Moraxella Catarrhalis*	1 (3.2)
Other identification	
*Mixed oral flora*	8 (25.8)

* *Multiple germ identification in a same patient led to a total percentage higher than 100*.

If one or more microorganisms were identified in the admission mini-BAL, the median α-amylase value (25 IU/L [IQR: 2–230]) was significantly higher than if a sterile culture was reported (2 IU/L [IQR: 0–43]—*p* < 0.01).

## Discussion

In this study, with a cut-point of 8.5 IU/L, α-amylase sensitivity and specificity to predict aspiration pneumonia within the first 5 days were, respectively 63 and 62%. TPR and FPR were respectively 46 and 77%. Rates were more efficient for predicting early aspiration pneumonia, with sensitivity, specificity, TPR, and FPR of respectively 76.5, 61, 32, and 91.5%. Our results suggest that bronchial α-amylase dosage is of moderate interest for predicting aspiration pneumonia after OHCA. However, interesting characteristics can be provided by α-amylase value, as demonstrated by a certain number of findings (11, 12, 14). Our work is the first to study bronchial α-amylase value in this specific context.

General characteristics of patients, causes of OHCA, length of stay, and mortality are in line with current data (5, 7, 16). Global management of such patients, including TTM, follows updated guidelines (1). Microbiologic results were similar to those found in patients admitted to ICU following cardiac arrest (3, 5).

In our study, aspiration pneumonia prevalence was 34%, consistent with recent results. The large variability of criteria used to define infectious pneumonia in the literature is somewhat responsible for a significant heterogeneity of prevalence, ranging from 22 to 61% (3, 7, 16). We decided to use both clinical and radiological criteria, and to consider pneumonia only if bacterial culture was proven within 5 days. Such restrictive conditions may explain the relatively low prevalence found. We did not consider the occurrence of fever or hypothermia because of the confounding factor of TTM, as well as the high incidence of hyperthermia on the following days of OHCA (17, 18). The common initiation of prophylactic antibiotic treatment on admission day (57% in our study) may also explain the lowered prevalence of microbiologic proof of pneumonia.

In the specific context of OHCA, the difference between aspiration pneumonia and ventilator-associated pneumonia (VAP) can lead to a certain confusion. Aspiration pneumonia is defined as the inhalation of either oropharyngeal or gastric contents into the lower respiratory tract. Acid gastric content can cause “chemical” injury, followed by an inflammatory pulmonary response, and eventually an infection (19). When infected secretions are the content of inhalation, they directly result in the infectious process. These two mechanisms are often involved. VAP is defined as pneumonia occurring more than 48 h after intubation and mechanical ventilation. Despite the protective role of the endotracheal tube, micro-aspirations occur and develop VAP (20). Even though these two mechanisms differ and are difficult to distinguish, the underlying physiopathology is similar.

The threshold of 8.5 IU/L identified in our work is lower than values reported in other studies. Samanta et al. (12) worked on bronchial α-amylase values in 151 patients undergoing mechanical ventilation, with suspected VAP. α-amylase concentration was significantly higher in patients with confirmed VAP. It was associated with the presence and number of inhalation risk factors. The 130 IU/L as cut-point had a sensitivity of 84% and a specificity of 67% for predicting VAP occurrence in patients with at least one risk factor of inhalation. The retrospective study of Weiss et al. (11) showed similar results. α-amylase dosage was performed on a blind or endoscopy-guided BAL. Ge-Ping Qu et al. (21) included 147 patients and reported that α-amylase concentration in tracheal samples of intubated patients was a good predictive value for VAP occurrence, with a sensitivity of 80% and a specificity of 79% (AUC 0.813) with a 4,681.5 IU/L threshold. The different sampling methods may explain the large variation of values described.

The population studied in our study may also explain the low median α-amylase value found. Our work focused on patients admitted to ICU after OHCA. In this specific context, inhalation can occur at the time of the cardiac arrest, following the loss of protective reflexes in the airway. Life support techniques (chest compression, mask ventilation) may also favor inhalation, until orotracheal intubation. This period is limited because intubation delay ranges from 10 to 20 mins following OHCA. Other studies focus on ICU patients, undergoing mechanical ventilation since a few days (11, 12, 21). Micro-inhalation events could also explain higher α-amylase values in those patients. Early dosage (within the first 6 h after admission) in our work also accounts for our lower values.

To our knowledge, there is no gold-standard technique to assess aspiration pneumonia. Numerous biomarkers of aspiration have been studied in patients undergoing mechanical ventilation or spontaneously breathing. All of them are of limited use in daily clinical practice. Pepsin is a powerful gastric enzyme. As it attests only gastric content, and poorly reflects oropharyngeal content, its interest remains incomplete (22, 23). Bile acid detection in tracheal samplings has also been studied, in small populations (24). Serum PCT validity in distinguishing bacterial and aspiration pneumonias is not yet proven (25). In the specific context of cardiac arrest, no biomarker seems adequate to predict aspiration pneumonia. α-amylase bears interesting characteristics: quantitative dosage is simple, of rapid response (a few hours), and inexpensive. Along with other clinical, biological, and radiological criteria, α-amylase dosage could help physicians decide when to initiate an antibiotic therapy.

The interest of prophylactic antibiotic therapy in preventing aspiration pneumonia is still debated (5–7, 16). In a controlled, randomized clinical trial, François et al. (7) studied the impact of a prophylactic treatment by amoxicillin-clavulanate during 2 days in patients admitted following OHCA with shockable rhythms. Patients' characteristics, management, and overall incidence of aspiration pneumonias were comparable with our study. Incidence of aspiration pneumonia at day 5 was lower in the treated group compared to the placebo group. Mechanical ventilation duration, ICU length of say, and mortality at day 28 did not differ. In our work, occurrence of aspiration pneumonia did not influence these outcomes either.

Our study has inherent limitations. The monocentric design and the small number of patients are undeniable. The population of interest is specific, and results may not be generalized to all suspected aspiration pneumonias in ICU patients. The lack of a gold-standard technique to assess aspiration pneumonia does not allow the comparison of our results. Despite a standardized sampling protocol, an inter-operator variability remains possible.

## Conclusion

Bronchial α-amylase value is not an effective biomarker for predicting aspiration pneumonia during the first 5 days following ICU admission after OHCA. The performances of this biomarker were higher but still insufficient for predicting early aspiration pneumonia (inferior to 48H). A systematic prophylactic treatment by amoxicillin-clavulanate during 2 days after ICU admission seems to be the better strategy.

## Data Availability Statement

The raw data supporting the conclusions of this article will be made available by the authors, without undue reservation.

## Ethics Statement

The studies involving human participants were reviewed and approved by Centre de protection des personnes sud méditérannée. The patients/participants provided their written informed consent to participate in this study.

## Author Contributions

JB and MG were responsible for study concept and design. AM, JB, JC, MG, FB, AL, SH, EC, AB, and JF were responsible for the acquisition, analysis, or interpretation of data. JB, EC, and AM were responsible for drafting the manuscript. MB and AM were responsible for statistical analysis. All authors had full access to all the data in the study, take responsibility for the integrity of the data and the accuracy of the data analysis, interpreted the findings, contributed to writing the manuscript, and approved the final version for publication.

## Conflict of Interest

The authors declare that the research was conducted in the absence of any commercial or financial relationships that could be construed as a potential conflict of interest.

## Publisher's Note

All claims expressed in this article are solely those of the authors and do not necessarily represent those of their affiliated organizations, or those of the publisher, the editors and the reviewers. Any product that may be evaluated in this article, or claim that may be made by its manufacturer, is not guaranteed or endorsed by the publisher.

## References

[1] Nolan JP Sandroni C Böttiger BW Cariou A Cronberg T Friberg H. European resuscitation council and european society of intensive care medicine guidelines 2021: post-resuscitation care. Intensive Care Med. (2021) 47:369–421. 10.1007/s00134-021-06368-4 33765189 PMC7993077

[2] Mongardon N Perbet S Lemiale V Dumas F Poupet H Charpentier J. Infectious complications in out-of-hospital cardiac arrest patients in the therapeutic hypothermia era. Crit Care Med. (2011) 39:1359–64. 10.1097/CCM.0b013e3182120b56 21336107

[3] Mortensen SJ Hurley M Blewett L Uber A Yassa D MacDonald M. Infections in out-of-hospital and in-hospital post-cardiac arrest patients. Intern Emerg Med. (2020) 15:701–9. 10.1007/s11739-020-02286-3 32052366

[4] Simons RW Rea TD Becker LJ Eisenberg MS. The incidence and significance of emesis associated with out-of-hospital cardiac arrest. Resuscitation. (2007) 74:427–31. 10.1016/j.resuscitation.2007.01.038 17433526

[5] Perbet S Mongardon N Dumas F Bruel C Lemiale V Mourvillier B. Early-onset pneumonia after cardiac arrest: characteristics, risk factors and influence on prognosis. Am J Respir Crit Care Med. (2011) 184:1048–54. 10.1164/rccm.201102-0331OC 21816940

[6] Gagnon DJ Nielsen N Fraser GL Riker RR Dziodzio J Sunde K. Prophylactic antibiotics are associated with a lower incidence of pneumonia in cardiac arrest survivors treated with targeted temperature management. Resuscitation. (2015) 92:154–9. 10.1016/j.resuscitation.2015.01.035 25680823

[7] François B Cariou A Clere-Jehl R Dequin P-F Renon-Carron F Daix T. Prevention of early ventilator-associated pneumonia after cardiac arrest. n Engl J Med. (2019) 381:1831–42. 10.1056/NEJMoa1812379 31693806

[8] Global Antimicrobial Resistance and Use Surveillance System (GLASS) Report: 2021 [Internet]. Disponible sur: Available online at: https://www.who.int/publications-detail-redirect/9789240027336 [Cité 13 Janv 2022].

[9] Centers for Disease Control and Prevention. Antibiotic Resistance Threats in the United States, 2019.—Recherche Google [Internet]. Available online at: Disponible sur: https://www.google.com/search?q=Centers+for+Disease+Control+and+Prevention.+Antibiotic+Resistance+Threats+in+the+United+States%2C+2019.&oq=Centers+for+Disease+Control+and+Prevention.+Antibiotic+Resistance+Threats+in+the+United+States%2C+2019.&aqs=chrome..69i57j69i60l2.7343j0j15&sourceid=chrome&ie=UTF-8 [Cité 11 Janv 2022].

[10] Dhital S Warren FJ Butterworth PJ Ellis PR Gidley MJ. Mechanisms of starch digestion by α-amylase-structural basis for kinetic properties. Crit Rev Food Sci Nutr. (2017) 57:875–92. 10.1080/10408398.2014.922043 25751598

[11] Weiss CH Moazed F DiBardino D Swaroop M Wunderink RG. Bronchoalveolar Lavage amylase is associated with risk factors for aspiration and predicts bacterial pneumonia. Crit Care Med. (2013) 41:765–73. 10.1097/CCM.0b013e31827417bc 23314582

[12] Samanta S Poddar B Azim A Singh RK Gurjar M Baronia AK. Significance of mini bronchoalveolar lavage fluid amylase level in ventilator-associated pneumonia: a prospective observational study. Crit Care Med. (2018) 46:71–8. 10.1097/CCM.0000000000002774 29053492

[13] Lorentz K. Approved Recommendation on IFCC Methods for the Measurement of Catalytic Concentration of Enzymes. Part 9. IFCC Method for Alpha-Amylase (1,4-Alpha-D-Glucan 4-Glucanohydrolase, EC 3.2.1.1). International Federation of Clinical Chemistry and Laboratory Medicine (IFCC). Committee on Enzymes. Clin Chem Lab Med. (1998) 36:185–203. 10.1515/CCLM.1998.034 9589808

[14] Torres A Niederman MS Chastre J Ewig S Fernandez-Vandellos P Hanberger H. International ERS/ESICM/ESCMID/ALAT Guidelines for the Management of Hospital-Acquired Pneumonia and Ventilator-Associated Pneumonia: Guidelines for the Management of Hospital-Acquired Pneumonia (HAP)/Ventilator-Associated Pneumonia (VAP) of the European Respiratory Society (ERS), European Society of Intensive Care Medicine (ESICM), European Society of Clinical Microbiology and Infectious Diseases (ESCMID) and Asociación Latinoamericana del Tórax (ALAT). Eur Respir J. (2017) 50:3. 10.1183/13993003.00582-2017 28890434

[15] Kalil AC Metersky ML Klompas M Muscedere J Sweeney DA Palmer LB. Management of adults with hospital-acquired and ventilator-associated pneumonia: 2016 clinical practice guidelines by the infectious diseases society of America and the American thoracic society. Clin Infect Dis Off Publ Infect Dis Soc Am. (2016) 63:e61–111. 10.1093/cid/ciw504 27418577 PMC4981759

[16] Hellenkamp K Onimischewski S Kruppa J Faßhauer M Becker A Eiffert H. Early pneumonia and timing of antibiotic therapy in patients after nontraumatic out-of-hospital cardiac arrest. Crit Care Lond Engl. (2016) 20:31. 10.1186/s13054-016-1191-y 26831508 PMC4736704

[17] Makker P Kanei Y Misra D. clinical effect of rebound hyperthermia after cooling postcardiac arrest: a meta-analysis. Ther Hypothermia Temp Manag. (2017) 7:206–9. 10.1089/ther.2017.0009 28731840

[18] Gebhardt K Guyette FX Doshi AA Callaway CW Rittenberger JC Post cardiac arrest service. prevalence and effect of fever on outcome following resuscitation from cardiac arrest. Resuscitation. (2013) 84:1062–7. 10.1016/j.resuscitation.2013.03.038 23619740

[19] Marik PE. Aspiration pneumonitis and aspiration pneumonia. n Engl J Med. (2001) 344:665–71. 10.1056/NEJM200103013440908 11228282

[20] Weinberger J Cocoros N Klompas M. Ventilator-associated events: epidemiology, risk factors, and prevention. Infect Dis Clin North Am. (2021) 35:871–99. 10.1016/j.idc.2021.07.005 34752224

[21] Qu G-P Fang X-Q Xu Y-P Shi M Wang Y Gong M-L. Predictive value of α-amylase in tracheal aspirates for ventilator-associated pneumonia in elderly patients. Clin Respir J. (2018) 12:1685-92. 10.1111/crj.12729 29087039

[22] Jaoude PA Knight PR Ohtake P El-Solh AA. Biomarkers in the diagnosis of aspiration syndromes. Expert Rev Mol Diagn. (2010) 10:309–19. 10.1586/erm.10.7 20370588 PMC2882092

[23] Nseir S Zerimech F Jaillette E Artru F Balduyck M. Microaspiration in intubated critically ill patients: diagnosis and prevention. Infect Disord Drug Targets. (2011) 11:413–23. 10.2174/187152611796504827 21679139

[24] Wu Y-C Hsu P-K Su K-C Liu L-Y Tsai C-C Tsai S-H. Bile acid aspiration in suspected ventilator-associated pneumonia. Chest. (2009) 136:118–24. 10.1378/chest.08-2668 19318678

[25] El-Solh AA Vora H Knight PR Porhomayon J. Diagnostic use of serum procalcitonin levels in pulmonary aspiration syndromes. Crit Care Med. (2011) 39:1251–6. 10.1097/CCM.0b013e31820a942c 21283001 PMC3102149

